# Validation of life-charts documented with the personal life-chart app – a self-monitoring tool for bipolar disorder

**DOI:** 10.1186/s12888-015-0414-0

**Published:** 2015-03-14

**Authors:** Lars O Schärer, Ute J Krienke, Sandra-Mareike Graf, Katharina Meltzer, Jens M Langosch

**Affiliations:** 1 Department of Psychiatry and Psychotherapy, University of Freiburg Medical Center, Freiburg, Germany; 2 Bethanien Hospital for Psychiatry, Psychosomatics and Psychotherapy, Gützkower Landstrasse 69, Greifswald, 17489 Germany

**Keywords:** Life-chart, Bipolar disorder, Validation, Self-monitoring, App, Quality of life

## Abstract

**Background:**

Long-term monitoring in bipolar affective disorders constitutes an important therapeutic and preventive method. The present study examines the validity of the Personal Life-Chart App (PLC App), in both German and in English. This App is based on the National Institute of Mental Health’s Life-Chart Method, the de facto standard for long-term monitoring in the treatment of bipolar disorders.

**Methods:**

Methods have largely been replicated from 2 previous Life-Chart studies. The participants documented Life-Charts with the PLC App on a daily basis. Clinicians assessed manic and depressive symptoms in clinical interviews using the Inventory of Depressive Symptomatology, clinician-rated (IDS-C) and the Young Mania Rating Scale (YMRS) on a monthly basis on average. Spearman correlations of the total scores of IDS-C and YMRS were calculated with both the Life-Chart functional impairment rating and mood rating documented with the PLC App. 44 subjects used the PLC App in German and 10 subjects used the PLC App in English. 118 clinical interviews from the German sub-sample and 97 from the English sub-sample were analysed separately.

**Results:**

The results in both sub-samples are similar to previous Life-Chart validation studies. Again statistically significant high correlations were found between the Life-Chart function rating assigned through the PLC App and well-established observer-rated methods. Again correlations were weaker for the Life-Chart mood rating than for the Life-Chart function impairment. No relevant correlation was found between the Life-chart mood rating and YMRS in the German sub-sample.

**Conclusion:**

This study gives further evidence for the validity of the Life-Chart method as a valid tool for the recognition of both manic and depressive episodes. Documenting Life-Charts with the PLC App (English and German) does not seem to impair the validity of patient ratings.

## Background

The course of bipolar disorder is often characterised by a multitude of individual, very diverse symptoms [1]. In most cases, drug combinations are necessary to cope with these symptoms [2,3]. Given the very large number of possible drug combinations, it cannot be expected to find studies in accordance with the criteria of “Evidence Based Medicine” for all combinations, however. Therefore, other sources of empirical evidence are needed as a basis for clinical decisions.

As with many other chronic illnesses, long-term monitoring in bipolar affective disorders (BD) constitutes an important therapeutic and preventive method. In the past, it has been demonstrated repeatedly that tools supporting self-management through the earlier recognition of a pending bipolar episode and increased drug compliance have a positive impact on the course of bipolar affective disorders [4,5]. Thus long-term monitoring appears to be a source of crucial empirical information as a basis for the optimisation of therapy.

### The National Institute of Mental Health’s Life-Chart Method (NIMH-LCM)

For the long-term documentation of BD in the form of self-assessment, several patient diaries have been developed in which patients judge their state of mood and the severity of manic and depressive episodes at regular intervals. One of these diaries is the National Institute of Mental Health’s Life-Chart Method (NIMH-LCM), which has become the de facto standard for long-term monitoring in the treatment of bipolar disorders.

The NIMH-LCM can be used to rate the past (“Retrospective Life-Chart”) or the present day by day (“Prospective Life-Chart”). There are clinician-rated and patient-rated forms of documenting the Life-Charts. Retrospective Life-Charts are documented on forms covering several years by month; Prospective Life-Charts are documented on forms covering one month, day by day. Combining this information, the self-rated prospective Life-Chart was used as a “patient diary”.

The variables documented with the NIMH-LCM include a bidirectional CGI-type Scale for functional impairment due to manic or depressive symptoms. The user chooses an interval between −4 to +4, with −4 standing for the maximum impairment due to depressive symptoms and +4 representing maximum impairment due to manic symptoms. Additionally, the lowest and highest mood of the day is entered on a bar chart or with values ranging between 0 and 100, with 0 signifying the lowest, most depressed mood, 50 indicating a balanced mood and 100 for a euphoric mood. Other entered parameters include the number of mood changes and the presence of dysphoric mania. For each administered drug, the brand name, dose with unit, number, pharmaceutical form, side effects and compliance are recorded, along with sleep duration, hospitalizations, menstruation, significant life events or cumbersome situations and any comorbid symptoms.

The extraordinary importance of the continuous, long-term monitoring of bipolar affective disorders has already been established through the use of the Life-Chart Method (LCM) for a long time now [6-8] The Validation of the clinician-rated prospective LCM was assessed previously. Mainly two studies provide evidence for the validity of Life-Chart ratings. The first study correlated clinician-rated Life-Charts with scores of the Inventory of Depressive Symptomatology and clinician-rated (IDS-C) and with scores of the Young Mania Rating Scale (YMRS) [9]. The second study correlated patient-rated Life-Charts with scores of the Inventory of Depressive Symptomatology, clinician-rated (IDS-C) as well as with scores of the Young Mania Rating Scale (YMRS) [10].

The Personal Life-Chart (PLC) originally named Palm Life-Chart Method (PLC) [11] is an App based on the NIMH-LCM [11]. Instead of documenting Life-Charts on paper, patients enter data into the electronic devices of their choice. The PLC App can be used with both mobile devices and personal computers connected to the PLC Cloud. The PLC App method is available in several languages.

### Current study

This study investigates the validity of Life-Charts documented with the PLC App in the English and German language versions. The validity analysis was carried out by comparing the PLC App with pen and paper forms of well-established psychometric scales.

The primary objective of this study was to investigate the supposed correlation between impairment of social function (“function”) and the score of the psychometric scales Inventory of Depressive Symptomatology, clinician-rated (IDS-C) [12,13] and Young Mania Rating Scale (YMRS) [14], both in the depressed and (hypo-)manic state. The secondary target was to examine the supposed correlation between self-rated mood and clinician ratings.

## Methods

### Study description

The method of the study was copied to a large extent from the two other published Life-Chart validation studies [9,10]. The substantial difference to these “template studies” is that in this study the Life-Charts were not documented with a PEN AND PAPER (P&P) Life-Chart form, but rather with an ELECTRONIC Life-Chart form, through the use of the PLC App. As with the template studies this study was a prospective, multi-site natural observation study. The validity analysis was done in the same way as in previous studies: Total Scores of the Inventory of Depressive Symptomatology, clinician-rated (IDS-C) and of the Young Mania Rating (YMRS) were cross correlated with Life-Chart ratings for functional impairment and mood.

The total study duration was 18 months for each sub-sample.

### Subjects

This study analyses two sub-samples: 44 German-speaking and 10 English-speaking subjects. Patient networks within the German society for bipolar disorders facilitated the recruitment of the German sub-sample. This recruitment focussed on the number of patients. The recruitment of the English sub-sample was facilitated by the overseas internships of a doctoral candidate.

Patients were contacted in outpatient departments, on ward or at events of the German society for bipolar disorders with an invitation document giving an overview over the study. Subjects interested in study participation received detailed information with the informed consent document.

The study was conducted under approval of the ethical review committee of the University Hospital, Freiburg, Germany (Amendment of 06.03.2003 to Approval Nr. 114/99).

Inclusion criteria for the study (for both sub-samples) were a) DSM-IV diagnosis of BD I or II, BD not otherwise specified or schizoaffective disorder of bipolar type b) use of one of the versions of the PLC App in German or English respectively c) 18 years old or older and d) the ability to understand the goal of the study and to give consent.

Diagnoses were ascertained by the study team testing for DSM IV Criteria with standardized questions (SKID) whereby an MD confirmed all diagnoses. Despite broader diagnostic inclusion criteria, only patients diagnosed with BD I or II participated in the study.

Subjects were recruited from users of the PLC App. Nevertheless to ensure sufficient experience in the use of the PLC App, all participants received a standardized training in the use of the PLC App. A minimum of two months of Life-Chart usage was required before Life-Chart data was used for the study.

If questions or technical problems arose, subjects were able to report this and received immediate assistance by phone or by personal communication. Study data was entered in accordance with data protection regulations.

### Procedures

#### The PLC App

The PLC App – for a more detailed description see [11] - is an electronic diary for patients with bipolar disorder. The rating period for the electronic diary is the one-day rating. Participants documented Life-Charts daily with the App. During the study, patients were able to record and edit data for the current and the previous day.

#### Clinical interviews and psychometric testing

Clinical interviews were used to assess the severity of manic and depressive symptoms. Psychometric Testing was done through the use of standardised questionnaires. The Inventory of Depressive Symptomatology, clinician-rated (IDS-C) [12,13] and the Young Mania Rating (YMRS) [14] were used to measure depressive and manic symptoms, respectively. The IDS-C rates depressive symptoms for a period of the previous 7 days. Some IDS-C items report the average symptom severity in that period (e.g. weight change). Most IDS-C items assess the maximum severity during that period. The YMRS rates manic symptoms within the last 48 hours.

The intervals between clinical interviews were 32 days on average. If patients were likely to show acute manic symptoms, they were contacted more frequently in order to capture the typically fleeting acute manic symptoms. The minimal interval between clinical interviews was 2 days, the maximum 205 days (median 23 days). Staff performed the clinical interviews personally or by phone due to the sometimes long distances to the outpatients. First clinical interviews with outpatients and clinical interviews with inpatients were always conducted in person, however. The staff was composed of assigned clinicians and doctoral candidates who successfully completed inter-rater reliability training. At the time of the clinical interviews, raters were blind to the Life-Charts patients entered into their devices.

#### Data collection

The subjects of the study provided Life-Chart data in the form of monthly Life-Chart reports printed from the PLC Cloud using personal computers.

**Figure 1:**
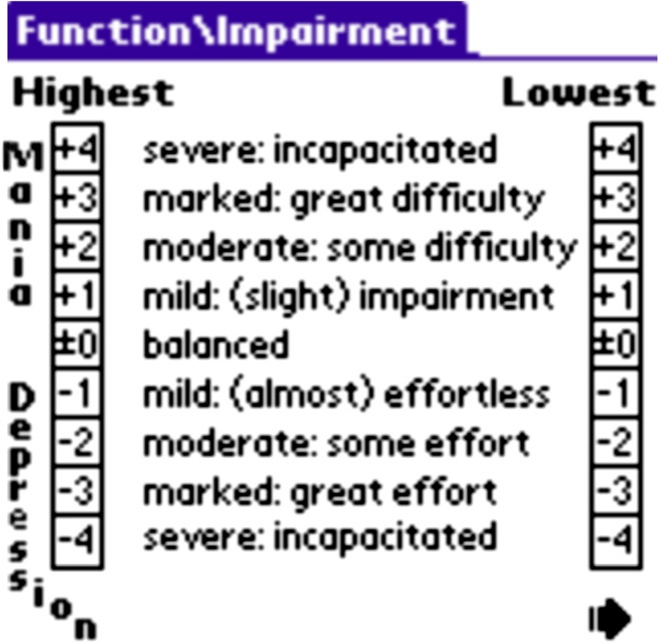
**Screenshot, English version.**

In the German-speaking sub-sample first generation mobile devices (Palm OS) were used for Life-Chart documentation. In the English-speaking sub-sample, a variety of devices were used: PDAs, Smartphones and web browsers were all utilised to document Life-Charts (Figure 1). One patient without internet access sent Life-Chart data to the PLC Cloud using coded text messages entered into a mobile phone.

The PLC App was used daily to document Life-Charts. Except for the browser solution and the one patient who entered data via text messages, Life-Chart data needed to be transferred from the PLC App to the PLC Cloud with a patient initiated synchronization process. The entire data collection process is not part of this study.

The data source of this study was the monthly provided Life-Chart reports, exclusively. The PLC App enables the integrated printing of the Life-Chart information documented day by day into these monthly Life-Chart forms which are almost identical with the NIMH Life-Chart used in the prior Life-Chart validation studies.

Using this feature, the subjects of the study provided Life-Chart data in the form of monthly Life-Chart reports printed from the PLC Cloud using personal computers.

### Data analysis

Thus data analysis was based on the paper versions of the Life-Charts – as with the template studies [9,10].

The assessed scores of the Inventory of Depressive Symptomatology, clinician-rated (IDS-C) and the Young Mania Rating Scale YMRS were correlated with the Life-Chart functional impairment ratings and mood ratings.

Furthermore the monthly Life-Chart report form closely resembles the paper Life-Chart forms used in the two aforementioned Life-Chart validation studies. This allowed for the use of exactly the same method for data analysis as with the template studies.

For the investigation of the validity of the PLC, Spearman Correlations were calculated. For calculation of the correlation between Life-Chart and IDS-C we examined the maximum depression scores (mood and “function”) documented on the Life-Chart within the 7-day period rated by the corresponding IDS-C. And in order to calculate the correlation between the Life-Chart and YMRS we used the maximum manic scores (mood and “function”) documented on the Life-Chart within the 48 hour period rated by the corresponding YMRS.

Life-Chart ratings outside of the assessment periods of IDS-C and YMRS ratings were not included in the study. Data was stored anonymously in MS Excel files and analysed with SPSS. Data access was limited to study staff, according to privacy protection regulations in Germany.

## Results

### Description of the sample

This study combines data from two sub-groups: data from 118 clinical interviews with 44 German-speaking subjects and from 97 clinical interviews with 10 English-speaking subjects plus the Life-Chart data of the subjects.

All participants in the German-speaking sub-sample were outpatients, 23 of them were male and 21 female; 39 subjects came from Germany, 3 subjects from Austria, one from Switzerland and one from Croatia. All were native German speakers. The subject’s average age was 40.6. On average 3 clinical interviews were conducted per patient.

In the English-speaking sub-sample 4 subjects came from Canada, 3 from South Africa and 3 from the USA. 6 of these native English speakers were male and 4 female, 8 in outpatient and 2 in inpatient care. At the beginning of the study, the subject’s average age was 34. On average, the patients’ onset of symptoms had begun 8 years before being correctly diagnosed. An average of 9 clinical interviews were carried out per patient.

### Most common symptoms

In the IDS-C the most common symptoms were sleep onset and mid-nocturnal insomnia, sadness, anxiety, impaired concentration and psychomotor agitation. In the YMRS, questions about elevated mood, increased motor activity, energy, irritability and an increase in the rate and the amount of speech, obtained the highest scores.

### Correlation of life-chart ratings with IDS-C scores

The Personal Life-Chart rating (PLC) of functional impairment by depressive symptoms highly correlates and statistically significantly with the IDS-C total score in the German and in the English-speaking sample (see Table 1). There is also a statistically significant correlation between IDS-C total scores and lowest mood in both groups.

**Table 1:** **Validation of life-charts documented with the personal life-chart app – A self-monitoring tool for bipolar disorder** [11]

		PLC German	PLC English	Denicoff et al. 2000 [ 9 ]	Born et al. 2014 [ 10 ]
IDS-C	vs. function	−0.73	−0.72	−0.79	−0.72
	vs. mood	0.62	0.60	n.a.	n.a.
YMRS	vs. function	0.53	0.61	0.66	0.49
	vs. mood	0.17	0.39	n.a.	n.a.

Correlation coefficients in this study (PLC English and German sub-samples) compared to previous life-chart validation studies.

### Correlation of life-chart ratings with YMRS scores

A high and statistically significant correlation was found between YMRS total score and Personal Life-Chart rating (PLC) of functional impairment by manic symptoms in both samples. The correlation between YMRS and the highest Life-Chart mood rating was less prominent and significant only in the English-speaking sub-sample, but not for the German sub-sample. Severely incapacitating manic symptoms, corresponding to a Life-Chart functional impairment rating of +4, were not recorded in this study.

## Discussion

The PLC App is derived from the NIMH-Life-Chart Method, the de facto standard for the long-term monitoring of bipolar patients. The NIMH-Life-Chart Method has been validated in its observer-rated version [9] as well as recently in its self-rated version [10]. This validation study replicates the methods and results of these studies (“template studies”). The Life-Chart function impairment rating was found to correlate highly and statistically significantly again with IDS-C total score for depression and YMRS total score for mania. The new aspect of this study is only, that an electronic Life-Chart form was used instead of the pen and paper Life-Chart forms used in the template studies. To our knowledge this is the first validation study for an electronic Life-Chart.

The aim of the PLC App is to provide a validated self-rating Life-Chart method which is available to everyone and thus to complement and in some cases even replace observer-rated methods.

With the PLC App, the patients themselves document their depressive and manic symptoms. Therefore, the PLC App is a self-reporting tool as opposed to observer-rated scales such as the IDS-C or the YMRS. Previous studies reported that patients recognised self-monitoring as useful [15]. The electronic methods seem to perform better than pen and paper methods (P&P) and patients favoured electronic diaries over P&P [16]. Additionally the validity of electronic diaries was found to be similar to the preceding P&P versions [17].

The results of this study confirm previous findings for P&P Life-Chart versions, too [9,10]. These template studies found high correlations between IDS-C and the Life-Chart function impairment ratings (r = −0.785, p ≤ 0.01 and r = −.718; p < .001). This is similar to the correlations found in this study (r = −0.726, ≤ 0.01). As in the template studies, the correlation for YMRS and Life-Chart Function impairment rating was slightly lower.

Life-Chart functional impairment rating with the PLC App predicts the severity of depressive symptoms diagnosed with IDS-C again very well. The high and statistically significant correlation between patient-rated Life-Chart functional impairment due to depressive symptoms and the IDS-C total score (−0.717 in the English speaking and r = −0.726 in the German speaking sample) give further evidence for the validity of the Life-Chart method as a tool for the documentation of both manic and depressive symptoms. Thus documenting Life-Charts with the PLC App does not seem to impair the validity of patient ratings.

With the still high and statistically significant correlation (Spearman correlation of 0.610 in the English-speaking sample and r = 0.531 in the German- speaking sample) between patient-rated functional impairment due to manic symptoms registered with the Personal Life-Chart and the YMRS scores, Life-Charts again demonstrate their potential for the recognition of manic symptoms. The slightly lower correlation in the mania range might be explained by reduced illness awareness at higher levels of mania for some patients, however.

As with the template studies the correlation between the IDS-C total score and Personal Life-Chart mood rating was weaker than for Personal Life-Chart functional impairment rating (0.6 versus 0.72). This difference was even more pronounced for YMRS versus Life-Chart function and mood ratings, respectively. In the German sub-sample the correlation coefficient was only 0.388 (Spearman) and in the English sub-sample even lower. The results in both sub-samples are very similar so that language does not seem to affect the validity of the ratings.

Some limitations have to be considered:

1. Severe mania hardly occurred in the patient sample, thus not a single value of +4 for functional impairment in the PLC App was obtained. Therefore, the investigation of the validity for self-rating of severe mania is not part of this study. And since insight into the illness is more frequently impaired while experiencing severe mania, it is plausible not to expect this validity. This does not impair the clinical value of Life-Chart methods, because severe mania is easy to recognise for those in the patient’s environment and usually leads to rapid treatment, however. As such, the key is to recognise lower levels of (hypo)mania.
2. The sample was probably representative regarding the most common symptoms and the duration of the symptoms until the correct diagnosis. Other than this, the sample was probably made up of a very diverse group of patients with different levels of insight, compliance, familiarity with devices, intensities of medical care etc. Each of these factors might influence the validity of the ratings. This reduces the likelihood of obtaining a statistically significant result, however. So obtaining a statistically significant result despite the heterogeneous group is actually evidence for the strength of effect. Nevertheless larger studies are needed in order to identify crucial factors for the validity of the self-ratings of individual patients.
3. Participants were already well trained in the use of this Life-Chart method when entering the study. Patients who are not familiar with Life-Chart forms (paper or electronic) might not be able to document valid Life-Charts.
4. The sample size in the English speaking sample was too small (n = 10) and Life-Chart documentation methods varied too much as to warrant generalizable results. However, as results in both sub-samples are very similar, there is no indication that language, duration or method of Life-Chart documentation affects the validity of the ratings. Nevertheless larger samples are required for validation and even larger ones to allow for a stratified analysis.
5. Feasibility suffered from the technical challenges of the first generation of Apps [18]. With the text message version, hardly any technical problems arose, but the non-intuitive syntax took time for some of the patients to get used to, however. A certain amount of technical knowledge on the patient’s side was therefore a prerequisite. Only a few years later, cloud technology and smartphones have become both more sophisticated and more feasible and users have become accustomed to both (Figure 2).

**Figure 2:**
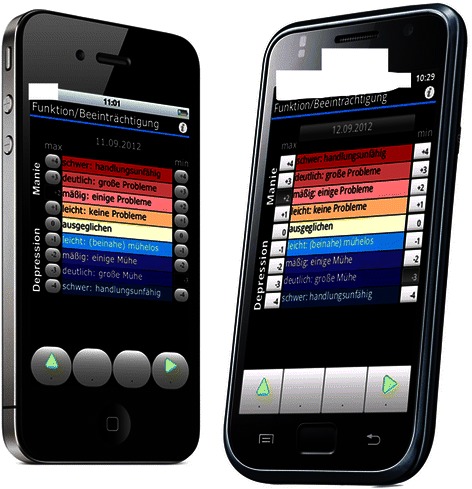
**Screenshot, German version, next generation.**
6. YMRS and IDS-C have not been validated for use in phone calls. However, clinical interviews by telephone reflect every day clinical practice and have been used in other studies. If they were to produce reduced measurement accuracy with IDS-C and YMRS, this would result in impaired correlations. So, again, finding high correlations despite this potential source of noise is an argument for the validity of the findings.

## Conclusion

This study provides further evidence for the validity of the Life-Chart method as a tool for the recognition of both manic and depressive episodes. Documenting Life-Charts with the PLC App does not seem to impair the validity of patient ratings, in this group of well-trained patients.

Thus the use of the PLC App on a daily basis can allow for continuous monitoring – also between clinical interviews – which might not be feasible with observer-rated scales already for financial reasons. With daily rating, detailed fluctuations can be illustrated, cyclical courses in terms of Rapid Cycling or Ultra Rapid Cycling can be recognised more easily and patients are more likely to remember their symptoms [11]. Compared to the pen and paper Life-Chart forms, electronic Life-Chart forms offer the advantage of less missing data; the automation of labour-intensive steps such as the continuous graphical analysis of the data saves resources; and both patients as well as clinicians profit from the immediate tabulation of the data [4,19].

Following the results of our and previous studies, the visual analogue scale mood rating is a) inferior to the bidirectional CGI-type functional impairment rating of the Life-Chart Method and b) a visual analogue scale mood rating might not be suitable for rating mania.

Electronic Life-Charts provided by the PLC-App can enable patients to validly assess the severity of depressive and manic episodes with functional impairment as well as with pen and paper Life-Charts. As a self-reporting tool this electronic diary can cost-effectively complement observer-rated methods such as the IDS-C or YMRS.

## Abbreviations

PLC: Personal life-chart
IDS-C: Inventory of depressive symptomatology, clinician-rated
YMRS: Young mania rating scale
BD: Bipolar affective disorders, bipolar disorders
NIMH-LCM: National Institute of Mental Health’s Life-Chart Method
LCM: Life-chart method
CGI: Clinical global impressions
